# Dr. Horace Green and the N. Y. Academy

**Published:** 1859-03

**Authors:** 


### EDITORIAL DEPARTMENT.

*Dr. Horace Green and the New York Academy.*—We have just received the February No. of the *American Medical Monthly*, which contains a full report of the investigations of the New York Academy of Medicine, into the case of Whitney, which was referred to in our last issue. We then commented upon the alleged conduct of Drs. Mott and Beales, as not in accordance with our views of professional honor. As we made this comment upon the authority of the New York *Times*, before hearing from the Medical Journals, we reproached ourselves, somewhat, for not having waited a full report; the authenticity, however, of the report in the *Times*, seemed undoubted. A full report has now appeared, which occupies about forty pages of the *Monthly*, and which we have carefully read. The distinguished reputation of Dr. Mott, earned by his meritorious labors in the cause of medical science, should make one hesitate to attack his reputation as a high-minded and honorable physician. It is due to ourselves to view his conduct in this affair with a charity which he did not show to Dr. Green, and we here only imitate the action of the Academy, which finally passed the following preamble and resolution, avowedly to shield the accusers, as well as to exonerate the accused:

"*Whereas*, Various statements made by the public press and otherwise, have reflected on the reputation of Dr. Green, and of Drs. Mott and Beales, as having conduced by their treatment to the death of Mr. Whitney; therefore

"*Resolved*, That we, the Academy of Medicine, after a full examination of the reports of the case, and the *post-mortem* examination, do consider that his death was in nowise the consequence of improper treatment, but was the unavoidable result of a complication of diseases."

This "*compromise*" was passed by nearly nine-tenths of the members present, and was afterwards made unanimous.

We have not sufficient space to publish the full report of the proceedings of the Academy, but we quote the analysis by Dr. Douglas, of the *Monthly*, with his comments, with which we cannot but agree.

A most unusual and unparalleled proceeding occupied most of the last three sessions of the Academy of Medicine. A gentleman, a son of one of the wealthiest of the citizens of New York, had been for two months a patient of a physician of this city, whose reputation is of both hemispheres. After one of the visits to this physician's office he was suddenly taken ill, and upon returning home, he sent for his former physician. Some alarming symptoms supervening, they were attributed to an operation performed by the first physician, and, from the social position of the family, the opinion of the second, at first cautiously expressed, perhaps, soon became a rumor, and passed through all the ramifications of society. The patient finally died, and in the mouths of the people, and even in the opinion of those of the profession, who had not heard the real facts of the case, his death was laid at the door of the first physician. The most ridiculous and unheard-of reports were circulated, till at last the rumor found its way within the walls of the Academy, and the hearing of the statements of all the physicians engaged in this case constituted the principal occupation of three of its sittings.

In this number of the Monthly will be found a full account of the transactions of the three sessions, as reported in the Times and Tribune. The accuracy of this report is unquestioned, and it must, therefore, stand as the actual proceedings of the Academy. It is now history, and will serve in the future to illustrate the condition of medical ethics and medical science in the city of New York, in the year 1859.

It is a pertinent question to ask, how came the public so interested in the death of this gentleman? A few words of explanation will add an introductory chapter to the already written history, and will show how soon, in this age of steam presses and eager reporters, everything which catches the ear of rumor is laid bare to the eye of the world.

We now invite the attention of our readers to the proceedings of the Academy, while we present this introductory chapter, for it is so connected with the history of the case, that, like the preface of a book, it is suggested by the case itself.

The statement by Dr. Green of the history of the case of Mr. Whitney, as long as he was under his care, tells its own story.

Then follows the statement made by Drs. Beales and Mott, with explanatory remarks by Dr. Beales. From this we learn that the excitement of the patient and his family was intense; that the patient attributed all his ills to the treatment received from Dr. Green; so positive was he of this, and so sure was the family of it, that had Dr. Green presented himself at the house he would have been met by personal violence. This feeling was not quieted by the family physician, but the excitement was permitted to express itself in violent criminations of Dr. Green. Fifteen hours after his last visit to Dr. Green, emphysema of the neck and face was observed, which gradually extended over a portion of the trunk. The patient was sure Dr. Green had injured his throat, and the emphysema was attributed to this injury. But how was the patient to know that any wound about the throat would produce emphysema? assuredly, only through his attending physician, who could account for it in no other way, and without looking for any other lesion, took the intemperate expression of the patient as evidence that the windpipe had been seriously injured. Here, then, was the source of the rumor of perforated windpipe; it was the physician's diagnosis, and no sooner expressed than it was taken up by a hundred tongues, and passed from mouth to mouth with the rapidity with which bad news always flies. Through the whole week of the patient's last sickness, this story gained credence. Each day's symptoms confirmed the previous day's rumor. At last the patient died, and a Sunday newspaper in chronicling his death, attributed it to the unskilful hand that had perforated the windpipe. Correspondents of papers, from without the city, taking up the hue and cry, which had then become loud and deep, sent abroad their news item, and back from city and village came the echo of death from punctured windpipe. The truth becoming known here, a flat denial was given by one of our city newspapers to this unfounded rumor. Here the matter rested till brought before the Academy.

With one single exception—that just referred to—the newspaper reports were all antagonistic to Dr. Green, so that the "suspicious source" of these rumors must be manifest to all who read the history of this case.

This only in the way of preface. We now come to the consideration of the case itself.

And first of Dr. Green's statement: Oct. 25, Mr. Whitney was examined and operated upon by Dr. Green.

Diagnosis: Tubercular softening under the left clavicle; throat granulated and inflamed; left tonsil slightly enlarged and ulcerated; epiglottis thickened, and its border whitened by a line of erosions. Treatment: "Enlarged and ulcerated portion of tonsil was removed; pharynx and sub-tonsillary fossæ, and the border of the eroded epiglottis, were cauterized."

This was the treatment pursued on the first day. Oct. 26 and 27, the local treatment by the probang was extended into the glottis. Nothing was done between the 27th October and the 9th November, when treatment was again renewed, to be interrupted till the 18th November. At both of these latter visits the local treatment was the same as that used on Nov. 27. On the 20th November another physical examination was made, and a diagnosis similar to that given on the 25th October was the result. Then followed an interruption of two weeks, when, on the 4th December, another application to the larynx was made by the sponge probang. Dec. 6th, the gum elastic tube was passed down the larnyx and trachea, "and a drachm of the nitrate of silver solution, of the strength of fifteen grains to the ounce, was injected into the left bronchus." Dec. 9th, patient called again; was better, with less cough and diminished expectoration. He desired the tube to be passed. and the injection to be made at this visit. It was not used for reasons stated by Dr. Green. The patient came back on the 14th, his last visit, when the sponge probang was used.

This ends Dr. Green's record. In it we find a diagnosis clear and distinct; a plan of treatment founded on that diagnosis, and consonant with Dr. Green's views.

The sponge probang was used at every visit, with one exception, (Dec. 6,) when the tube and injection was used, much to the patient's satisfaction and relief.

Here commences the record of Drs. Beales and Mott. It is prefaced by Dr. Beales with a distinct avowal of antagonism to Dr. Green, and with a controversial animus, which should be foreign to the discussion of such a subject.

Reviewing the history of the case, as furnished by Drs. Beales and Mott, we find no diagnosis, no evidence that the nature of the difficulty under which the patient suffered was known to the attending physicians; but we do know that their diagnosis was perforated windpipe, from the reports which, as we have already shown, came from the family, and eventually found their way into the daily papers, and were carried into all parts of the country. We know it too from the post-mortem examination, for this perforation was sought for by them, and they expected to find it; a red point in the larynx, a little below the left chorda vocalis, led them to exclaim: "There is the point of laceration of the mucous membrane, by which the air has escaped into the cellular tissue to constitute the emphysema." On close inspection, and wiping the part with the sponge, no abrasion nor aperture could be discovered.

In all the history, we find no record of any examination of the chest having been made, no suspicion of any lesion in that region, no note of any remarkable characteristic in the expectoration; in fact, no evidence whatever that their attention had been directed to the lungs by the symptoms of the patient.

On Dec. 17th, and following days, he is said to have had considerable *mucous* secretion, which interrupted his respiration and gave him great trouble to expectorate, and was referred to the throat.

Impressed with the idea of a punctured windpipe, they could discover no other cause for the emphysema, which appeared early in the morning of the second day, fifteen hours after the last visit to Dr. Green.

Nor do we find any diagnosis of any special lesion in the throat, until the 19th, when we read from the record that "it is certain there is some serious lesion in the vicinity of the glottis," though the character of that lesion is not stated. That lesion they supposed to be a laceration of the windpipe, as we have shown. Early in the morning of the 21st the patient died, according to their record, "partly from exhaustion, partly by asphyxia."

From this we pass to the post-mortem examination. An abscess was found "about the size of a large hen's egg, and extending a little in front of the pharynx, and downward and below the thyroid cartilage. At the upper and posterior part of this abscess there was an opening into the pharynx, large enough to admit the end of the forefinger." The larynx and trachea were declared to be "natural and healthy," without "an abrasion." In the lungs, "just at the root," an open cavity about the size of a small walnut, of a reddish brown color and irregular villous surface, as though a slough had separated. At the upper and anterior part of this cavity, there was a small opening through both pleuræ.

The upper lobe of the left lung was mostly in a state of hepatization, and the pleuræ lying over this part of the lung was covered with "soft, *strumous-like* fibrin," which at first glance was taken for white thick pus.

Two prominent lesions, therefore—an abscess in the pharynx, and a cavity in the left lung, neither of which had been discovered or suspected during life, and a healthy and uninjured windpipe, when a punctured windpipe had been supposed to exist.

In connection with this point, we will simply draw attention to the singular incompleteness of the autopsy. Neither the cartilages of the larynx, nor the vertebræ, nor the intestines, nor any other organ, save the lungs and the internal surface of the larynx and trachea alone, were examined. Yet upon this meagre examination, a positive opinion is expressed.

Pursuing this analysis further, but not in the order in which the history of the case was given at the Academy, we reach the certificate of death given by the physician in ordinary, with the advice and consent of the consulting physician, viz.: *Effusion into the lungs.* The certificate given is a sufficient commentary on itself.

This completes the history of the case. In order to regard it from all points, we now propose to take up some of the statements made by Dr. Beales in his additional remarks, and to contrast them with the facts, as given in this short resume, and with the statements of his colleague. We shall then draw our conclusions therefrom.

About the end of October, the patient, who was then under the treatment of another physician for tubercular disease of the lungs, was examined by Dr. Beales, who pronounced his lungs free from tuburcles. Here, then, is an error of diagnosis, either on the part of Dr. Green or Dr. Beales. We have seen that Dr. Beales not only made an error of diagnosis, in the case already related, but failed to discover the former lesions which were the cause of the severe symptoms observed in the case of Mr. Whitney, and which eventuated in his death. A cavity, too, is found in the lungs, just at the po nt designated by Dr. Green at his first examination, (Oct. 25,) nearly itwo months previously. If this, then, is a tuburculous cavity, Dr. Green's diagnosis was correct, and Dr. Beales' wrong. That it was a tuberculous cavity Dr. Beales denies, and states his reasons, drawn from the post-mortem In reply to this Dr. Green quoted an extract from Rokitansky's Patholo gical Anatomy, giving the exact description of this form of tubercular cavi.ty.

The epiglottis, Dr. Green said, was thickened and eoroded at the first examination, and it was cauterized. At the post-mortem it was found "extraordinarily healthy, and free from the slightest vestige of disease." We must conclude from this that the treatment must have been very favorable in its effects. But Dr. Beales thinks otherwise, and says that under the circumstances "I am forced to believe that Dr. Green erred in his diagnosis, and that these various operations were unnecessary and uncalled for." We hardly think this comes with a good grace from one who has fallen into an error similar to that he charges upon another.

Pursuing our reading, we find in the next sentence that Dr. Beales avows his ignorance of the effects of nitrate of silver on the substance of the lungs, and yet he volunteers an opinion as to its effects, before closing the paragraph. If he will study the proceedings of the Paris Academy of Medicine, he will find that the very operation which he condemns as "at all times attended with extreme peril and risk of the patient's life," is safe, easily performed, and in daily use by the principal physicians of Paris, and commended by such as Trousseau, Depaul, Velpeau, and others, in the treatment of some diseases of the air-passages in children. If he will read the periodical medical literature of our own country, he will find that the probang is passed daily into and through the larynges of children with the greatest impunity, and with the effect of saving lives in those diseases where, before the introduction of this method of treatment, the mortality was almost certain. That a solution of the crystals of nitrate of silver, of the strength of 15 grains to the ounce of water, produced an ulceration of the lungs which resulted in a slough, no one, who knows the effects of the solution of the crystals of nitrate of silver, would assert.

That a slough or eschar could have been made at the "root" or apex, or superficies of the lung, by the injected fluid, and the patient exhibit no symptoms of any kind to warrant this assertion during the period of fifteen days which intervened between the operation and his death, we cannot believe. Eight days of this period he was apparently as well as he had been for several months, and even better than usual, according to his own statement.

The position of this "cavity" or "eschar," is badly defined. In the post-mortem it is stated to be "at the root, or at the commencement of the bronchial ramifications;" while Dr. Beales designates it in one place "as a shallow depression or scooping out of the actual apex or superficies of the lung," and in another place, "a slough or eschar at the apex of the lung." We cannot reconcile these conflicting statements.

There are some facts in connection with this point of the case which were not presented to the Academy, and which may have some influence, both in illustrating the character of the cavity in the lung, and account for the abscess in the pharynx. For that reason we will introduce them here.

It is known that the injection into the lung was made December 6th. The patient after this was better. It is also known that on the 12th of December he drove a pair of horses some distance into the country on Long Island, dined with a relative, and returned to New York in the evening by the same conveyance. It was remarked that day by his friends that he appeared in better health and spirits than they had known him to be for many months. At this very time, according to Dr. Beales, a destructive sloughing of the lung was progressing, and yet no expectoration, no pain, in fact, no symptom to indicate it.

It seems to us, that this drive into the country was the starting point of the abscess in the pharynx, which was in its incipiency on the 14th, and therefore the whole pharynx was sensitive to the application of the probang, and yet it was not sufficiently developed to attract attention. This abscess, Dr. Beales says, has been called a *chronic* abscess, and he calls Dr. Green to account for so naming it. We find no record in the whole transaction that this has been so called by Dr. Green or any of his friends, and we conclude that it is, therefore, only a "man of straw" which Dr. Beales has ingeniously set up for the purpose of demolishing, as he attacks this abscess most unmercifully—*on paper.*

Dr. Beales makes another charge against Dr. Green, that on the 14th December "the pharynx was accidentally lacerated by the probang." Into this laceration, says Dr. B., "doubtless portions of the various foreign bodies he attempted to swallow, food and medicine, were forced," and as a result, "sloughy abscess." We find no mention in the post-mortem that any portion of food or medicine the patient had taken were found in this laceration, and "doubtless" this is a mere hypothesis of Dr. Beales, and founded upon the same basis as his assertion relative to the effects of nitrate of silver upon the substance of the lungs.

It would be well to inquire, before making such an assertion, if an instrument introduced into the throat, through the mouth, could be brought into contact with this portion of the pharynx with force sufficient to produce a laceration of the mucous membrane, however slight. For our part, we do not believe it is possible, and the opinion expressed by Dr. Mott would seem to sustain it; for he said, in the debate which ensued, that speaking "anatomically, that abscess could not be reached *per orem*—through the mouth;" and in another place, "that any man, knowing the anatomy of the pharynx and larynx, would say immediately that that abscess could not have been got at by the fauces, so as to have been opened." It seems strange to us. then, how Dr. Beales could account for its being produced in the manner he suggests; for if an instrument could reach it to produce it, another assuredly could reach it to open it.

We believe that the theory—for theory alone it is—which Dr. Beales has erected is incorrect, unsubstantial, and without a shadow of a foundation.

There are other incongruities in the remarks of Dr. Beales, which we shall also point out, for the reason that we believe great injustice has been done Dr. Green by the first false reports, which could have been quieted, had the attending physician been so disposed to do—and because these incongruities will show the animus with which Dr. Beales closes his remarks in the following language: "This is all I think it needful to say in answer to these unmerited and disgraceful inuendoes."

And first, of the abscess in the pharynx. This abscess was filled with pus and destroyed filamentous tissue. It was confined by the deep, dense cervical fascia, and yet so full as to give a remarkable prominence to the thyroid cartilage. How this was possible, with a hole in the abscess as large as the end of the forefinger, is beyond our comprehension. The pressure of the parts alone, not taking into account the horizontal position of the patient, would cause the contents of the abscess to seek an exit wherever the opening might be, while the horizontal position would have favored this flow, had there been an opening there previous to the death of the patient. We regard this statement as prima facie evidence, that the opening was a post-mortem one, as all who are in the habit of making necroscopic examinations know how easy it is to make accidental incisions when removing hollow organs, and how difficult it is to avoid tearing their walls.

In order "doubtless" to make his case a very strong one, Dr. Beales says that "not the slightest sign of any chronic disease in or about the lung was found;" and further states what was supposed to be unanswerable, "that so striking was this fact, that Dr. Mott told the family after the post-mortem examination that we had not seen any disease that might not have been produced within a week." This, therefore, releases the operation of the catheterism and injection from any participation in the disease of the lung, or places Dr. Mott and Dr. Beales in a position of antagonism; for the operation referred to was performed fifteen days before the death of the patient, and eight before the commencement of his fatal illness.

Another point upon which Dr. Mott and Dr. Beales are at variance, refers to the question of medical ethics and medical courtesy. At the sitting of the Academy of the 4th of January, Dr. Mott said, "that he had proposed, as had Dr. Beales, that Dr. Green should be called in during the progress of this case, which was not assented to by the family." At the sitting of January 19th, Dr. Beales says that "had Dr. Green shown any sympathy for the family, &c., it would have afforded an opportunity to Dr. Mott and myself to have introduced him. Under the circumstances, it was no pleasant thing to ask permission of the family, and I frankly allow, *we* did not."

Many other incongruities and conflicting statements could be pointed out, but these are quite sufficient to show their value, and to point the moral, which may be drawn from Dr. Beales' closing sentence, already quoted.

Altogether, this is a most curious case in its developments; and those of our readers who have followed us through the analytical argument we have made, from the very words used by the parties, will agree with us, that it was a most unusual and unprecedented proceeding. The unscientific and incorrect certificate of death—the excuse given, that of desire to shield Dr.

Green, when all the harm that could reach him had already been accomplished by the thousand vague reports, is a most singular development of the customs and manners of the profession here.

To us the case seems perfectly clear. We do not believe that the lesion in the pharynx, or that in the lung, could, in any manner, be attributed to the operations of Dr. Green. The abscess in the pharynx might, and probably would, have occurred had Mr. Whitney never seen Dr. Green, nor had his throat operated upon. Several of a similar character, and in nearly the same location, occurred in this city at the same time. The cavity in the lungs, we believe, was a tuberculous cavity, situated near the surface of the lung, surrounded by tuberculous hepatization, and, by a singular coincidence, it burst in an access of coughing into the pleural cavity, causing collapse by the effusion of pus and air into the pleural cavity. From the pleural cavity the air, after a certain time, gradually forced its way through the opening in the costal pleura, at the point where the two pleuræ had been slightly adherent, and thus the time intervening between his first collapse and the appearance of the emphysema can be accounted for. This not an unusual occurrence, and is, therefore, not a mere hypothetical case.

The opening in the abscess of the pharynx, we believe, to have been a post-mortem opening, for we find no evidence either in the history of the symptoms, or the progress of the case, to warrant us in entertaining the idea that it existed there before.

There are a few thoughts, as regards the effect of such a discussion both upon the profession and the public, with which we should like to close our remarks. As, however, we have already taken up considerable space with the subject, we shall defer these to another time, should there be occasion to return to it.

The foregoing remarks were prepared before the last meeting of the Academy, as a resumé of its two previous sittings. We have delayed issuing this number of the Monthly, in order to complete the proceedings, which were continued at the first meeting in February. For the purpose of accomlishing this, we have added sixeen pages to our usual issue.

The last sitting of the Academy offers nothing essentially new to remark upon, except the singular method of argumentation employed, and which may be formularized thus:

The abscess in the pharynx was first an abscess which could not have been discovered; and if it had, it could not have been opened; and if it had been opened, it would have been attended with no beneficial results.

Again, this abscess is filled with pus and broken-down filamentous tissue. Then it is no longer an abscess, but a *cavity*, filled with a little pus and disintegrated cellular tissue; and, finally, it is an empty cavity, with a *little pus* at its lower portion, and disintegrated filamentous tissue about its sides.

The lesion in the lung has also its protean characteristics. It is first a cavity, and then a mere scooping out of the superficies of the lung; first at the root, and then at the apex of the lung.

Before finally dismissing this subject, we have one word to say, relative to Dr. Watson's theory. We believe it to be untenable, from his own showing. We take him at his word, that the lesion in the lung was of long duration; not of a week, nor a month's standing. Why should it be the result of gangrene? Because the patient was in the habit of drinking to excess in early life; because he had a cough and foul breath. This is insufficient ground for such a theory, particularly when we learn, as Dr. Watson states, that several members of his family had died of tubercular disease of the lungs. The reply to Dr. Watson's question relative to the medical treatment employed by Dr. Green, is another reason for us to doubt the correctness of Dr. Watson's theory. It is evident that there was a cachexia established in this patient, which very frequently terminates in tubercular disease. There is no necessity, then, for seeking for an unusual occurrence to account for his symptoms. The foul breath (which was not remarked before) and cough can be accounted for far more easily than by supposing a gangrene of the lung. From the history of the patient, then, from the history of his symptoms, and from the post-mortem appearances even, the inevitable conclusion to our minds is, that the lesion of the lung was a tubercular lesion. J. H. D.

